# Nutritional Intake after Liver Transplant: Systematic Review and Meta-Analysis

**DOI:** 10.3390/nu15112487

**Published:** 2023-05-26

**Authors:** Lynsey N. Spillman, Angela M. Madden, Holly Richardson, Fumiaki Imamura, Danielle Jones, Marilyn Nash, Hong Kai Lim, Holly N. Hellawell, Kirsten L. Rennie, Linda M. Oude Griep, Michael Allison, Simon J. Griffin

**Affiliations:** 1 MRC Epidemiology Unit, School of Clinical Medicine, University of Cambridge, Cambridge CB2 0QQ, UK; fumiaki.imamura@mrc-epid.cam.ac.uk (F.I.); dlj36@medschl.cam.ac.uk (D.J.); kirsten.rennie@mrc-epid.cam.ac.uk (K.L.R.); linda.oudegriep@mrc-epid.cam.ac.uk (L.M.O.G.); profgp@medschl.cam.ac.uk (S.J.G.); 2 Liver Transplant Unit, Cambridge NIHR Biomedical Research Centre, Cambridge University Hospitals NHS Foundation Trust, Cambridge CB2 0QQ, UK; michael.allison6@nhs.net; 3 School of Life and Medical Sciences, University of Hertfordshire, Hatfield AL10 9AB, UK; a.madden@herts.ac.uk (A.M.M.); hollyjrichardson@gmail.com (H.R.); 4 Department of Nutrition and Dietetics, East Suffolk and North Essex NHS Foundation Trust, Colchester CO4 5JL, UK; 5 School of Clinical Medicine, Addenbrooke’s Hospital, University of Cambridge, Cambridge CB2 0QQ, UK; hongkai.lim@doctors.org.uk (H.K.L.); hnh23@cam.ac.uk (H.N.H.); 6 Department of Public Health and Primary Care, Primary Care Unit, School of Clinical Medicine, University of Cambridge, Cambridge CB2 0SR, UK

**Keywords:** liver transplant, diet, nutrition

## Abstract

Cardiovascular disease and its concurrent risk factors are prevalent after liver transplant (LT). Most of these risk factors are modifiable by diet. We aimed to synthesise the literature reporting the nutritional intake of liver transplant recipients (LTR) and the potential determinants of intake. We performed a systematic review and meta-analyses of studies published up until July 2021 reporting the nutritional intake of LTR. The pooled daily mean intakes were recorded as 1998 (95% CI 1889, 2108) kcal, 17 (17, 18)% energy from protein, 49 (48, 51)% energy from carbohydrates, 34 (33, 35)% energy from total fat, 10 (7, 13)% energy from saturated fat, and 20 (18, 21) g of fibre. The average fruit and vegetable intake ranged from 105 to 418 g/day. The length of time post-LT and the age and sex of the cohorts, as well as the continent and year of publication of each study, were sources of heterogeneity. Nine studies investigated the potential determinants of intake, time post-LT, gender and immunosuppression medication, with inconclusive results. Energy and protein requirements were not met in the first month post-transplant. After this point, energy intake was significantly higher and remained stable over time, with a high fat intake and low intake of fibre, fruits and vegetables. This suggests that LTR consume a high-energy, low-quality diet in the long term and do not adhere to the dietary guidelines for cardiovascular disease prevention.

## 1. Introduction

Clinical guidelines recommend high energy and protein intakes to aid recovery after a liver transplant (LT) and to avoid foods that may cause food-borne infections, but dietary guidelines after the initial recovery phase are lacking [1]. Cardiovascular disease causes 19% of non-hepatic deaths after LT. The main risk factors for post-transplant death include post-transplant diabetes and hypertension [2]. Cardiovascular disease risk factors are prevalent in liver transplant recipients (LTR): 67–87% are overweight or obese, 41–63% have hypertension, 21–45% have diabetes, and 31–70% have dyslipidaemia [3,4,5,6,7,8,9,10,11,12]. These risk factors are modifiable by changes in lifestyle, including diet [13]. Systematic reviews of studies in general populations have found that plant-based and Mediterranean-style diets, and diets that are high in fruits, vegetables and whole grains and low in saturated fat are associated with reduced rates of premature all-cause mortality, cardiovascular mortality, cardiovascular events and cancer mortality [14,15,16,17,18,19].

Understanding dietary behaviours and the potential determinants of diet after LT can inform the development of evidence-based behavioural interventions and dietary guidelines that aim to improve health outcomes after LT. To our knowledge, no systematic review of published data concerning the diet or nutritional intake of LTR has been published previously. In this review, we aimed to synthesise the literature reporting the nutritional intake of LTR and the potential determinants of intake and to investigate the causes of heterogeneity in reported intake between studies.

## 2. Materials and Methods

We followed the preferred reporting items for systematic reviews and meta-analyses (PRISMA) guidance when preparing this manuscript [20]. The protocol of this systematic review was registered in the international prospective register of systematic reviews (PROSPERO ID: CRD42019126884).

### 2.1. Study Selection

We included studies with any study design that reported nutritional intake, or the factors associated with intake, among LTR of ≥18 years old. We excluded qualitative studies, studies reported in the form of an abstract only, and studies not published in English.

### 2.2. Search Strategy

We searched the Cochrane Library, MEDLINE, Embase, AMED, CINAHL, PsycINFO, BNI, Web of Science and OpenGrey electronic databases from the earliest date available until 4 July 2021, as a part of a wider literature review regarding the dietary and physical activity behaviours of LTR. The search strategy is presented in Table S1 (Supplementary Materials). The reference lists of all the included studies and relevant reviews were screened to identify additional studies.

### 2.3. Study Selection

We collated the results of the literature search in Endnote X8 (Clarivate, Philadelphia, PA, USA). We then exported the deduplicated results to Rayyan for screening. Two reviewers (LS and DJ/MH/HR) independently screened the titles, abstracts and full-text articles. We resolved any disagreements through consultation with a third reviewer (SG).

### 2.4. Data Extraction

Two reviewers (LS and MH/HH/HK/HR) extracted all the data independently using piloted data extraction forms. We extracted the following data: study design and location, demographic and clinical characteristics, sample size, dietary assessment method, dietary/nutritional intake, and potential behaviour determinants. We extracted the average and variance values for energy, protein, carbohydrate, fat, saturated fat, fibre, dietary quality scores and food groups, including fruits and vegetables. We extracted data in any format reported in the papers, at any time post-transplant, and recorded the time after the transplant took place. For the nutritional intervention studies, we extracted the dietary results at baseline for both groups (intervention and control) and the follow-up for the control group only. We attempted to contact the corresponding authors of the papers up to three times to request any missing information.

### 2.5. Risk of Bias

Two reviewers (LS and MH/HH/HK/HR) independently assessed the included articles for risk of bias (ROB) using the Critical Appraisal Skills Programme (CASP) checklist for cohort and cross-sectional studies and the Cochrane risk of bias tool, plus two additional questions from the CASP checklist (‘Was the sample recruited in an acceptable way?’ and ‘Was the diet accurately measured to minimise bias?’) for intervention studies [21,22]. We resolved disagreements by consulting a third reviewer (SG).

### 2.6. Data Synthesis and Analysis

We reported the potential dietary behaviour determinants, food group intake and diet quality score using narrative methods, due to the limited availability of studies or heterogeneity in terms of study methods. First, we converted energy (kcal/day) and macronutrient (percentage of energy/day) intakes into mean and standard deviation (SD) where necessary. We also converted the median and interquartile range (IQR) or range of intakes to arithmetic means and SDs, for which we assumed a median and IQR, followed a log-normal distribution and used the algorithm previously reported [23]. Some studies reported various combinations of means, SDs, standard errors, ranges, changes from a study baseline and *p*-values for changes. We harmonised these data into means and SDs using the approaches documented in the Cochrane Handbook [24]. We assumed that the ranges of intake equated to four SDs. Finally, we converted the macronutrients reported as grams per day using the Atwater conversion factors: 4 for protein, 4 for carbohydrate and 9 for fat [25].

We converted the intake reported as grams per kilogram per day or kilocalories per kilogram per day to total daily intake, using the study-specific average weight of the participants. If weight was not reported, we used the average of the weights reported from the other included studies. In the case of intervention studies, we pooled the baseline group means and SDs from the study arms [22].

We undertook the meta-analysis using Stata 16 (Stata Corp LLC, College Station, TX, USA). We pooled the mean daily energy and percentage of energy from the macronutrients and grams of fibre via random effects meta-analysis, using the DerSimonian and Laird method for pre-defined time categories, based on the recovery trajectories (<1 month, 1 to <6 months, 6 to <12 months and ≥12 months post-transplant). When a single study contributed to more than one estimate in a single meta-analysis, we treated the multiple estimates as independent after imputing the wider standard errors if two estimates had been independent [26]. We used I^2^ and Cochrane’s Q tests to examine the heterogeneity between estimates. We assessed the publication bias visually using a funnel plot for the mean energy intakes, stratified by time and based on heterogeneity in terms of intake by time post-transplant.

We conducted a series of bivariate random-effect meta-regression analyses to explore the associations between nutritional intake (total energy and percentage of energy from macronutrients) and study-level variables, including the average time post-transplant, average age, the proportion of males, the proportion of subjects with alcohol-related liver disease, continental area of the study, and dietary assessment method. The variables for meta-regression were selected a priori in terms of data analysis. We did not perform meta-regression for socioeconomic status or ethnicity because of the small number of estimates available (<10).

We undertook sensitivity analyses, excluding those studies with a high or unclear risk of bias for either of the following items, using the CASP risk of bias tool: (1) was the sample recruited in an acceptable way? and (2) was the diet accurately measured to minimise bias? [21] Additional sensitivity analyses were undertaken to compare data that required conversion and data that did not require conversion.

## 3. Results

**Figure 1:**
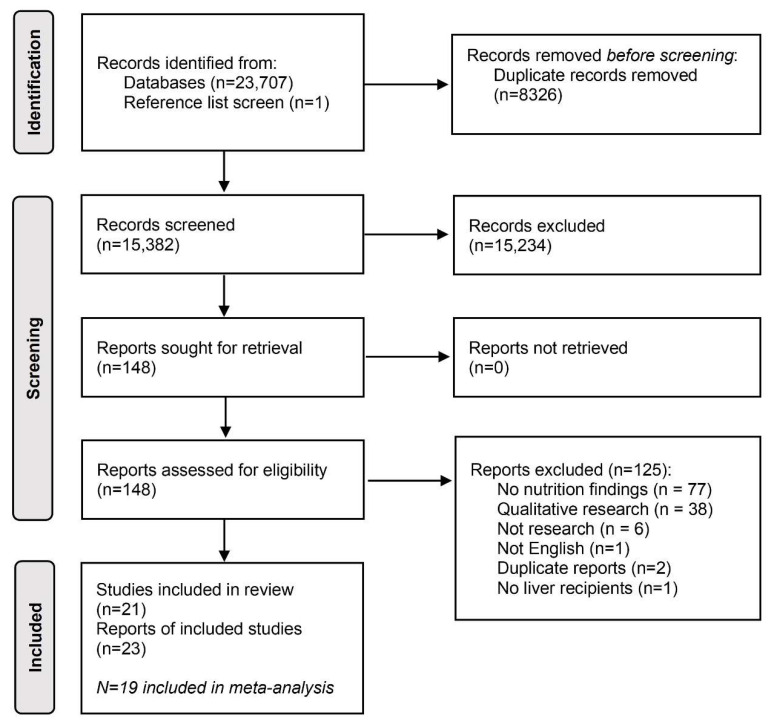
PRISMA (2020) flow diagram of studies that were identified, screened, excluded and included in the review.

Figure 1 presents the search and screening results from all the databases accessed. The characteristics of all 21 of the included studies (23 publications) are shown in Table 1. The studies were published between 2001 and 2021. Two were randomised control trials, thirteen were prospective cohort studies and six were cross-sectional studies. A total of 1150 patients participated. Nutritional intake was reported in 3, 7, 9 and 11 studies for ≤1 month, 1 to <6 months, 6 to <12 months and ≥12 months post-transplant, respectively. The average age ranged from 48 to 56 years, and 35–88% of the participants were male. Food diaries (*n* = 11), 24-h recall (*n* = 5), dietary history (*n* = 4), hospital food records (*n* = 1), food frequency questionnaire (*n* = 1) and the Mediterranean diet adherence screener (*n* = 1) were used for dietary data collection. Two studies used a combination of two dietary assessment methods [27,28,29] (Table 1).

### 3.1. Nutritional Intake

#### 3.1.1. Narrative Synthesis

Four studies categorised dietary intake in terms of food groups (Table S2, Supplementary Materials) [9,27,29,43]. It was inappropriate to pool the averages for fruit and vegetable intake as a conversion to arithmetic means and SDs produced implausible values. Two studies could not be included in the statistical synthesis: De Carvalho et al. (2010) reported mean daily energy intake as 33 kcal/kg and protein intake as 1.5 g/kg, but reported no variance [36]; Hickman et al. (2021) measured nutritional intake using the Mediterranean diet adherence screener (MEDAS), with a possible score ranging from 0 to 14 points, wherein higher scores indicated greater Mediterranean diet adherence. The mean (SD) MEDAS score was 6.0 (2.1), 5.9 (3.2) and 5.3 (1.4) for the control group at baseline, the control group at a twelve-week follow-up and the intervention group at baseline, respectively [39].

#### 3.1.2. Meta-Analysis

**Figure 2:**
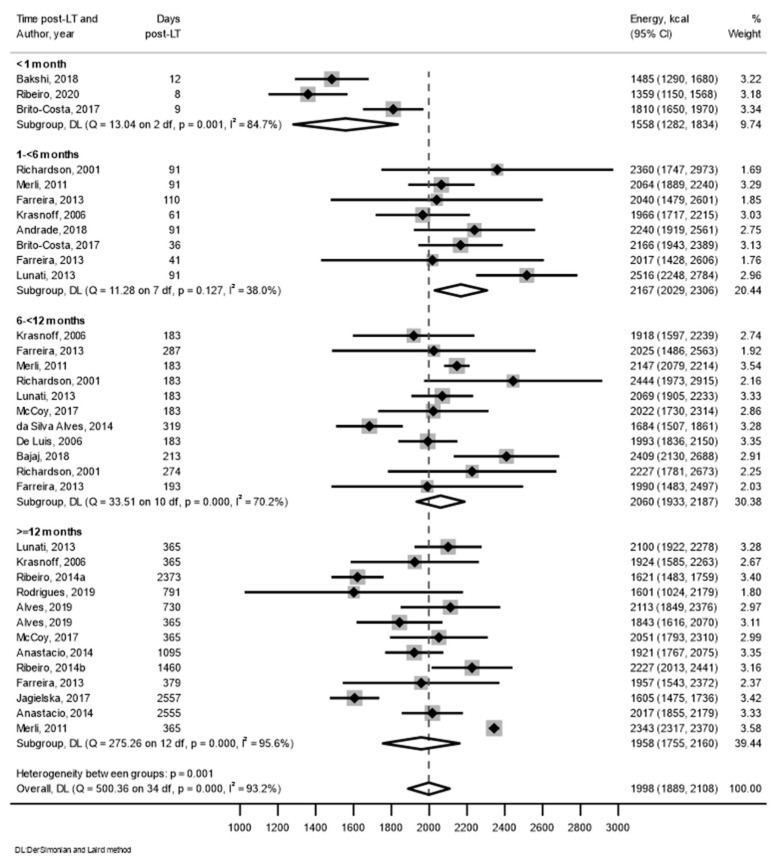
Mean daily energy intake (kcal/day) with 95% confidence intervals from the included studies, categorised by time post-transplant. CI: Confidence interval; DL: DerSimonian and Laird; LT: liver transplant. Data from references [3,4,9,28,29,30,31,32,34,35,37,38,40,41,42,43,44,45,46].

The results from 19 individual studies were available for a meta-analysis of the mean intake for energy (Figure 2), percentage of energy from macronutrients and grams of fibre (Figures S1–S5, Supplementary Materials). Nutritional intake is presented as forest plots in categories of months post-transplant and for overall intake (Figure 2 and Figures S1–S5). In the first month post-transplant, the recorded mean (95% CI) energy intake was 1558 (1282, 1834) kcal/day, with 19 (18, 20)% of energy from protein, 47 (44, 50)% of energy from carbohydrate and 34 (32, 37)% of energy from total fat. No studies reported fibre or saturated fat intake for the first month post-transplant. At 1 to <6 months post-transplant, the mean energy intake was 2167 (2029, 2306) kcal/day, with 17 (16, 19)% of energy from protein, 49 (47, 52)% of energy from carbohydrate, 33 (30, 36)% of energy from total fat, 7 (−1, 15)% of energy from saturated fat and 21 (19, 23) g of fibre. At 6 to <12 months post-transplant, the mean energy intake was 2060 (1933, 2187) kcal/day, with 17 (15, 19)% of energy from protein, 49 (46, 52)% of energy from carbohydrate, 35 (32, 37) % of energy from total fat, 10 (9, 12)% of energy from saturated fat and 21 (18, 24) g of fibre. At ≥12 months post-transplant, the mean energy intake was 1958 (1755, 2160) kcal/day, with 17 (16, 19)% of energy from protein, 50 (48, 52)% of energy from carbohydrate, 34 (32, 35)% of energy from total fat, 10 (9, 11)% of energy from saturated fat and 19 (18, 21) g of fibre.

### 3.2. Potential Determinants of Nutritional Intake

#### 3.2.1. Narrative Synthesis

Nine studies reported potential determinants of intake, including the time post-transplant (*n* = 8), sex (*n* = 1) and immunosuppression medication (*n* = 1). There was no difference in nutritional intake between participants taking cyclosporine when compared to tacrolimus [33]. Compared to males, fewer females met the guidelines for intakes of carbohydrates (<50% of energy) and fewer males met the guidelines for intakes of total fat and saturated fat (<30% of energy and <10% of energy, respectively). No sex difference was found regarding intakes of energy, protein or fibre [40]. Studies that investigated nutritional intake at different times post-transplant showed mixed results; as time progressed post-transplant, four studies reported an increase in energy intake [28,29,33,34,45], two studies reported an increase in grams of protein [29,45], one reported a reduction in the percentage of energy from protein [28], two studies reported a reduction in the percentage of energy from carbohydrates [33,34], one study reported increased grams of carbohydrate [43], and one study reported increased grams of fibre [28]. For all other reports of nutrient intake, there was no statistically significant difference over time (Table S3, Supplementary Materials).

#### 3.2.2. Meta-Regression

Table 2 shows the meta-regression results regarding time post-transplant. Energy intake was significantly lower in the < 1 month time period post-transplant, compared to all other time periods: 1 to <6 months (609 kcal/day mean difference (MD) 95% CI 292, 926 kcal/day, *p* = 0.002), 6 to <12 months (251 kcal/day MD, 95% CI 100, 402 kcal/day, *p* = 0.003), and ≥12 months (135 kcal/day MD, 95% CI 24, 247 kcal/day, *p* = 0.021). There were no significant differences in intakes of total energy or macronutrients between the periods after the first month post-transplant.

Table 3 shows the results from the meta-regression of other variables. There was no evidence that nutrient intakes varied among those participants with alcohol-related liver disease compared to those with other causes of liver disease. Energy intake appeared higher in studies with participants of a higher average age. Sex differences were not evident, except for the percentage of energy from saturated fat: saturated fat intake was higher in studies with a higher proportion of male participants. Overall, studies in Europe reported higher energy and percentage of energy from total fat and a lower percentage of energy from protein than those in other continents. Compared to older studies, recently published studies reported lower energy intakes with a higher percentage of energy from protein and a lower percentage of energy from total fat. There was no significant difference in nutrient intake by dietary assessment method (Table 3).

### 3.3. Risk of Bias (ROB)

**Figure 3:**
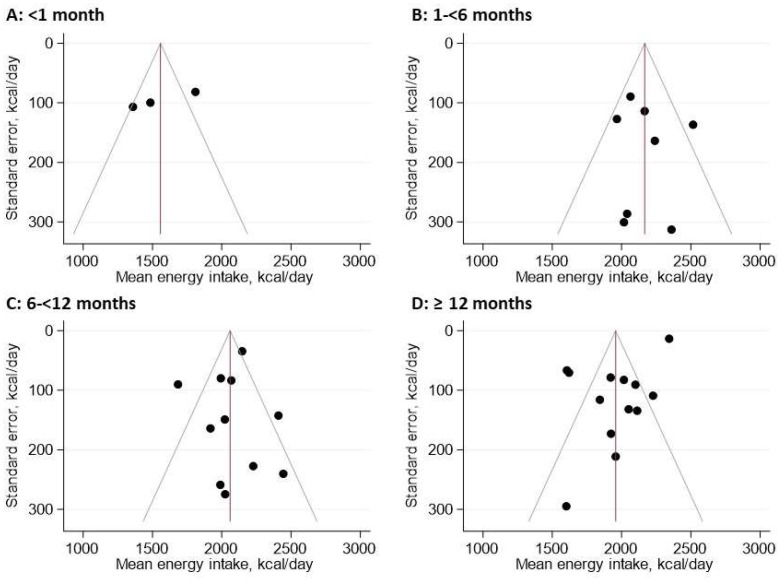
Funnel plot of daily energy intake (kcal). Due to the observed heterogeneity according to the study period, this funnel plot was stratified by period: <1 month, 1 to <6 months, 6 to <12 months, and ≥12 months.

**Figure 4:**
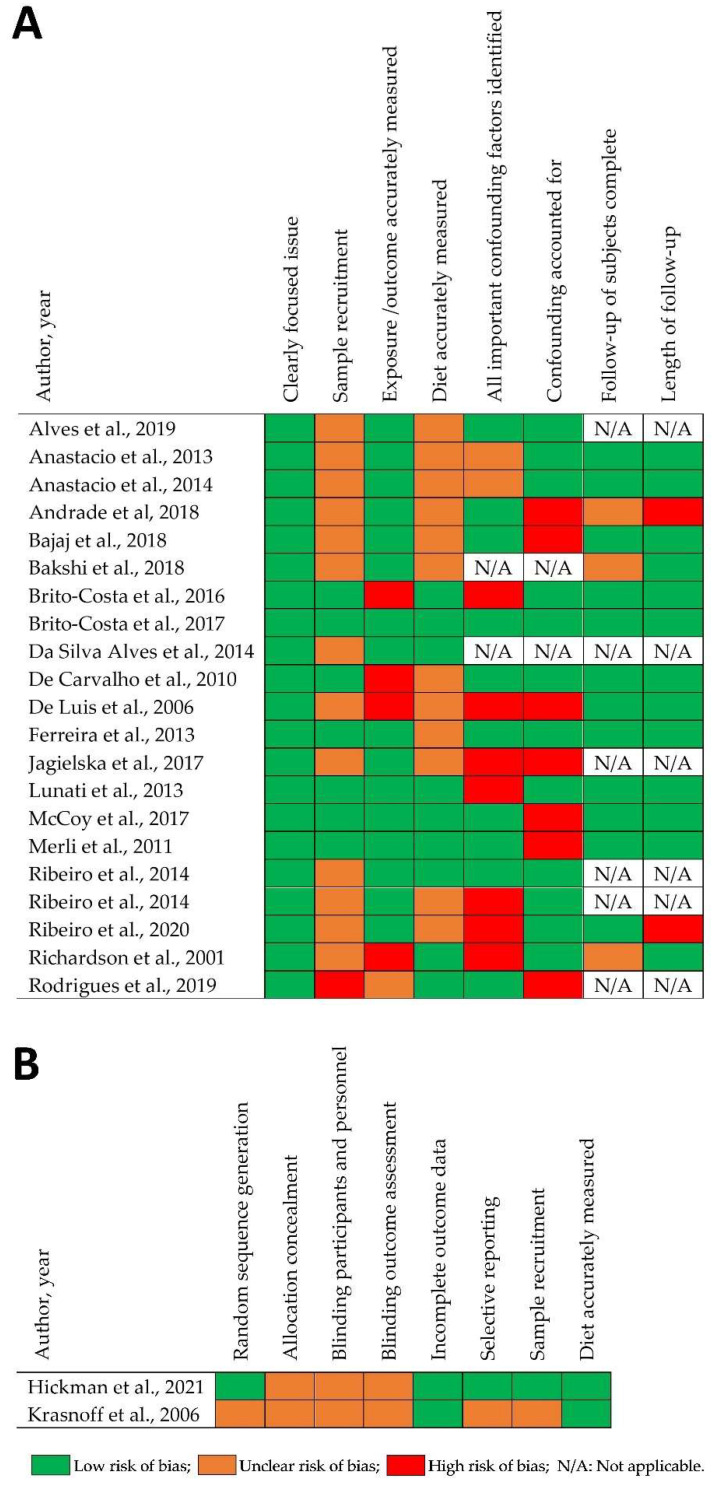
Risk of bias for 23 articles reporting 21 included studies: (**A**) cohort and cross-sectional studies, and (**B**) randomised control trials. N/A: Not applicable. Data from references [3,4,9,27,28,29,30,31,32,33,34,35,36,37,38,39,40,41,42,43,44,45,46].

Visual inspection of the funnel plot suggests that there is little indication of publication bias (Figure 3). The ROB assessment is shown in Figure 4. One study scored low in terms of ROB for all domains [34]. When the two key CASP questions were considered to assess the ROB for sensitivity analysis, namely, ‘Was the sample recruited in an acceptable way?’ and ‘Was diet accurately measured to minimise bias?’, five independent studies were assessed as having a low risk of bias. Four could be included in the ROB sensitivity analysis, providing a total of ten observations for meta-regression.

### 3.4. Sensitivity Analysis

#### 3.4.1. Risk of Bias

There were differences between the pooled means of studies with a low ROB compared to the pooled means without the exclusion: energy, fibre, total fat and saturated fat intakes were higher and carbohydrate intakes lower (Figures S6–S11, Supplementary Materials). The results of the meta-regression analyses including only those studies with a low ROB were unremarkable (Table S4, Supplementary Materials), with the exception of the average energy from protein, which was 5.0% higher (95% CI = 1.3%, 8.7%, *p* = 0.014) in European cohorts than in those from other continents, doubling the effect size in comparison to the meta-regression without the exclusion.

#### 3.4.2. Converted Data

Between 23% and 45% of the data required statistical assumptions to harmonise the estimates of intake. There was a significantly higher percentage of energy from total fat shown in the converted data when compared to data not requiring conversion (3.4% higher, *p* = 0.018, 95% CI 0.6, 6.2%). Energy, other macronutrients and fibre intake did not show any evidence of sensitivity to the harmonisation process (Table S5, Supplementary Materials).

## 4. Discussion

The findings of this systematic review and meta-analysis suggest that, on average, energy and protein recommendations for post-transplant recovery are not adhered to. Energy intake increased after one month post-transplant, but we found no significant difference in energy intake between 1 and 6, 6 and 12, and >12 months post-transplant. This suggests that patients consume a diet high in energy for longer than is required, which potentially leads to surplus energy intake and excess body weight and fat mass. Compared to international and national recommendations for general populations, we found that, on average, LTR generally consume a diet high in total fat and low in fibre, fruits and vegetables [47,48,49,50]. Time post-transplant, the average age of participants, the proportion of male participants, the continent of study and the year of publication were sources of heterogeneity in the meta-regression.

Nine studies investigated potential determinants of intake, of which eight examined the associations between time post-transplant and dietary intake. The findings were mixed, with studies reporting higher, lower or stable nutrient intakes as time progressed post-transplant. According to our meta-regression evaluating the findings from 19 studies, we found that energy intake was significantly lower at <1 month post-transplant compared to the three other time periods investigated. All studies reporting dietary intake for <1 month described the provision of nutrition support (oral, enteral or parenteral), following clinical protocols. One study reported energy and protein intakes from the diet, oral nutritional supplements (ONS) and parenteral nutrition (PN); only 14% of the energy intake came from ONS and 2% came from PN, suggesting a limited impact of nutrition support in this study [29]. The results from a retrospective study indicate that an enteral nutrition support protocol intervention helps to meet energy and protein requirements in the first week post-LT [51]. Our findings suggest that average energy and protein intakes are below recommended levels during the first month of post-transplant recovery; there is a need for the careful monitoring of nutritional intake and the provision of effective nutrition support to assist acute post-transplant recovery. 

For post-transplant time points beyond one month, we found no significant difference in energy intake. Studies that have investigated energy intake, expenditure, and body composition 3–12 months after transplant show an overall positive energy balance with weight and fat-mass gain [38,44]. After LT, many patients gain excessive weight and develop overweight issues or obesity. Studies have reported that 85% of LTR gain > 10% of body weight post-transplant, with an average weight gain of 9.5–11.6 kg at 3 years post-transplant and weight gain above their pre-morbid weights [5,52,53]. Obesity is an important risk factor for cardiovascular disease, which is common after LT [54]. The findings of the current review provide further evidence that patients consume a high-energy diet for longer than required, with low dietary quality. Both energy intake and physical activity are important modifiable risk factors to be targeted after a transplant. Studies using objective measurement methods found low levels of physical activity in LTR compared to the guidelines, which also influences energy balance [40,55,56,57]. 

It remains unclear for how long a high-energy and high-protein diet is required after a transplant, which warrants further investigation. This period may differ between individual patients with different causes of liver disease because LT is medically and surgically complex; nutritional status, post-transplant recovery and complications vary between individuals [58]. There are currently no post-transplant dietary guidelines that include recommendations regarding overall dietary quality, or guidelines for the post-recovery phase. A meta-review of systematic reviews in the literature found that guidelines for general populations are not implemented in patients with comorbidities as they are insufficiently tailored to the complex needs of these patients [59]. There is a need for the development of evidence-based dietary guidelines that are tailored to LTR, for example, with recommendations to aid recovery, treat malnutrition and sarcopenia, and promote long-term health, including weight management and cardiovascular risk reduction. Dietary guidelines specific to LT may help to better integrate diet as part of post-transplant care and better support clinicians’ communication with patients about this topic.

In this review, we found that LTR do not meet the dietary recommendations for healthy eating or cardiovascular disease prevention in terms of total fat, fruits, vegetables and fibre [47,48,49,50]. There is a paucity of research investigating overall diet quality for LTR: we identified only one study that assessed diet quality using a Mediterranean diet adherence score (MEDAS) and reported low scores of 5.3–6.0 out of a maximum of 14 points without intervention [39], while four studies reported the intake of food groups, noting average fruit and vegetable intake below recommended levels [9,27,29,43]. Traditionally, nutrition research has focused on a single nutrient or food; however, this approach fails to consider the importance of patterns of overall dietary intake on health outcomes. Nutrients are not eaten in isolation but as combinations of foods with complex interactions; therefore, no single element of a diet can provide a complete picture of the effect of diet on health [60]. Hence, dietary guidelines now focus on dietary patterns, rather than on individual nutrients or foods [49]. Validated diet quality scores assess the health impacts of dietary patterns [61]. Further diet-quality research and translation to clinical practice is needed for the LTR population.

The reasons for energy imbalance and poor diet quality post-transplant are unknown. Several factors may contribute to this tendency: appetite improves as illness resolves; pre- and immediate post-transplant high-energy intake to treat or prevent malnutrition may continue; there may be a return to the usual pre-illness behaviours or local prevailing intake norms; medication may affect appetite; inadequate psychological coping strategies may influence eating behaviours. Understanding the reasons for dietary behaviour through further qualitative and quantitative research will inform the design of future interventions to promote healthy eating.

To our knowledge, there are no published studies that have investigated the impact of diet on health outcomes, such as morbidity and mortality, in LTR. Three diet and physical activity intervention trials found improvements in the intermediate markers for health outcomes in LTR. In a dietary intervention, Pinto et al. tested adherence to the Brazilian guidelines for atherosclerosis prevention (low total fat, saturated fat and cholesterol intakes) on lipid profiles in 53 LTR who were 47 months post-transplant and found significant improvements in total cholesterol, low-density lipoprotein and triglycerides compared to pre-intervention levels [10]. In another dietary intervention study, adherence to the National Cholesterol Education Programme (energy-balanced, low fat and high fibre intakes) was tested and showed significantly greater improvements in health-related quality of life for the intervention, compared to the control group in 119 LTR who were recruited at 2 months post-transplant [41]. A Mediterranean-style diet telehealth intervention, delivered to 35 LTR four years post-transplant, resulted in the participants significantly decreasing their waist circumference, BMI and metabolic syndrome severity scores and improving their mental health-related quality of life [39]. This evidence highlights the finding that diet quality is an important area of improvement for LTR and that adherence to the general dietary guidelines for CVD prevention does achieve a reduction in risk factors in individuals post-transplant. Further research on dietary quality and the intakes of specific food groups, along with their impact on health outcomes, will help to identify more targeted dietary advice for LTR.

Narrative results from this systematic review identified gender as a potential determinant of nutritional intake consistent with the finding from our meta-regression that studies with more male participants reported greater energy intake from saturated fat. Gender is also a potential determinant of nutritional intake in other populations [62]. Future interventions need to target or be adapted for both males and females. We also found that the year of publication and the continent under study were sources of heterogeneity for some nutrients. Energy intake and the percentage of energy from total fat decreased and the percentage of energy from protein increased as the year of publication increased. This may be due to the growing recognition over time of the need for a healthy diet post-transplant [63]. Energy intake and the percentage of energy from total fat was higher and the percentage of energy from protein was lower in studies undertaken in Europe compared to those in other continents. This ecological finding should be interpreted with caution, to promote further research. Future cumulative evidence may lead to region-specific post-transplant dietary recommendations, accounting for variability in the patients’ anthropometry, underlying diet quality, and many other geological and sociocultural factors [64].

This systematic review has several strengths. Due to the systematic search that was conducted, this paper provides a comprehensive overview of the research investigating the dietary intakes of LTR, highlighting the strengths and limitations of research in this area and the need for future research. We have used robust methods to explore sources of heterogeneity to understand the differences between studies. It is also important to interpret the findings of our review within the context of the limitations. One study, including 211 LTR, which may have been eligible was excluded as it was reported in Korean [65]. We also identified two abstracts that we were unable to include as they were not published as the full text and further information could not be provided by the authors [66,67]. Further evidence eligible for inclusion may also have been published since the literature search; therefore, we re-ran the systematic search to identify evidence published between July 2021 and May 2023. The titles, abstracts and full-text manuscripts of the resultant documents were screened by a single reviewer. This identified one study that would be eligible for meta-analysis: Bahari et al. measured the dietary intake of 39 LTR in Iran at one and three months post-transplant. The total energy intake was lower than in the studies included in this review, with a similar percentage of energy from macronutrients [68]. The funnel plot gives little indication of publication bias, due to small studies. The statistics reported were heterogeneous between studies and the validity of pooling estimates with various statistical assumptions is a limitation; however, sensitivity analysis found no evidence of sensitivity to the harmonisation of data for all nutrients, except in the case of total fat. Studies included in this review did not account for transplant-related complications or illness, therefore study samples may not represent the diversity seen in the health and recovery of LTR. Only one study was rated as being at low risk of bias for all domains. An unclear risk of bias due to poor reporting is an issue with some of the differences in findings noted for studies with a lower risk of bias, compared to the findings from all studies. There is limited information about dietary assessment methods within the included study reports, making it challenging to assess the validity in detail or to establish how the evaluation methods differed for hospitalised compared to free-living participants. We recommend that future dietary research includes good-quality dietary assessment methods based on set guidelines and better reporting of methods [69]. We planned to assess heterogeneity in terms of socio-economic status, ethnicity and further aetiologies of liver disease; however, this was not possible due to missing information and inconsistent reporting.

## 5. Conclusions

In conclusion, the findings from nineteen studies investigating nutritional intake after LT suggest that energy and protein requirements for recovery are not met in the first month post-transplant. Following the acute recovery phase, energy intake was higher and stable between 1 and >12 months post-transplant. LTR consume, on average, a diet high in total fat and low in fibre, fruits and vegetables, and do not meet the general population guidelines for healthy eating or cardiovascular disease prevention. Potential determinants of dietary intake are not well studied, but the limited evidence suggests that time post-transplant, gender and geographical location are associated with nutritional intake. There is a paucity of evidence regarding overall dietary patterns and diet quality for LTRs.

## Figures and Tables

**Table 1:** Characteristics of the 21 studies included in the systematic review.

Author, Year *	Country	Study Design	Participant Details	Time Post-Transplant	Dietary Assessment Method
Alves et al., 2019 [30]	Brazil	Cross-sectional	*n*: 69 Median age (IQR): 51.6 (51–64) Males (*n*): 42 Females (*n*): 27	Median (IQR) Normal CIMT: 1 (4–9) year Abnormal CIMT: 2 (1–4) years	3-day food diary
Anastacio et al., 2013 [27]; 2014 [28]	Brazil	Prospective cohort	*n*: 148 Median age (range): 51.5 (21–75) Males (*n*): 90 Females (*n*): 58	Median (range) Baseline: 3 (0–13) years Follow-up: 7 (3–17) years	Diet history +/− 3-day food diary
Andrade et al., 2018 [31]	Brazil	Prospective cohort	*n*: 23 Age range: 18–65 Males (*n*): 11 Females (*n*): 12	3 months	3 24-h recalls
Bajaj et al., 2018 [32]	USA	Prospective cohort	*n*: 40 Mean (SD) age: 56 (7) Males (*n*): 35 Females (*n*): 5	Mean (SD): 7 (3) months	1 24-h recall
Bakshi and Singh, 2018 [29]	India	Prospective cohort	*n*: 54 Mean (SD) age: 48.6 (10.2) Males (*n*): 39 Females (*n*): 15	Range: 1–12 days	Hospital records + 2 24-h recalls
Brito-Costa et al., 2016 [33]; 2017 [34]	Portugal	Prospective cohort	n: 56 Mean age (SD): 53.7 (8.5) Males (*n*): 49 Females (*n*): 7	Median (IQR): Time 1: 9 (7–12) days, Time 2: 36 (31–43) days	1 24-h recall
Da Silva Alves et al., 2014 [35]	Brazil	Cross-sectional	*n*: 36 Mean (SD) age: 53.3 (10.6) Males (*n*): 22 Females (*n*): 14	Range: 1–24 months	1 24-h recall
De Carvalho et al., 2010 [36]	France	Prospective cohort	*n*: 70 Mean (range) age: 47.5 (23–69) Males (*n*): 41 Females (*n*): 15	45, 90, 180 and 365 days	3-day food diary
De Luis et al., 2006 [37]	Spain	Prospective cohort	*n*: 31 Mean (SD) age: 56.2 (8.1) Males (*n*): 25 Females (*n*): 6	6 months	3-day food diary
Ferreira et al., 2013 [38]	Brazil	Prospective cohort	n: 17 Mean (range) age: 52 (29–65) Males (*n*): 12 Females (*n*): 5	Mean (SD): Time 1: 41 (20) days Time 2: 110 (26) days Time 3: 192 (14) days Time 4: 287 (24) days Time 5: 379 (96) days.	3-day food diary
Hickman et al., 2021 [39]	Australia	Randomised (feasibility) controlled trial	*n*: 35 Mean (SD) age, intervention: 50 (15), control: 51 (15) Males (*n*): 25 Females (*n*): 10	Median (IQR): 4 (2–6) years	Mediterranean diet adherence screener
Jagielska et al., 2017 [40]	Poland	Cross-sectional	*n*: 44 Mean (SD) age: 51.5 (11) Males (*n*): 31 Females (*n*): 24	Not reported	7-day food diary
Krasnoff et al., 2006 [41]	USA	Randomised control trial	*n*: 151 Mean (SD) age, intervention: 49.5 (11.3), usual care: 50.6 (11.3) Males (*n*): 47 Females (*n*): 72	2, 6 and 12 months	The Block 95 full-length dietary questionnaire
Lunati et al., 2013 [42]	Italy	Prospective cohort	*n*: 84 Mean (SD) age: 53.9 (9.3) Males (*n*): 63 Females (*n*): 21	3, 6 and 12 months	3-day food diary
McCoy et al., 2017 [43]	Australia	Prospective cohort	*n*: 17 Median (IQR) age: 54 (16) Males (*n*): 14 Females (*n*): 3	6 and 12 months	Diet history
Merli et al., 2011 [44]	Italy	Prospective cohort	*n*: 25 Median (range) age: 55 (21–64) Males (*n*): 19 Females (*n*): 6	3, 6 and 12 months	Diet interviews
Ribeiro et al., 2014 [4]	Brazil	Cross-sectional	*n*: 42 Mean (SD) age: 50.1 (13.1) Males (*n*): 22 Females (*n*): 20	Mean (range): 6.5 (1.1–15) years	3-day food diary
Ribeiro et al., 2014 [9]	Brazil	Cross-sectional	*n*: 136 Mean (SD) age: 52.2 (13) Males (*n*): 83 Females (*n*): 53	Mean (SD): 4 (3) years	Diet history
Ribeiro et al., 2020 [45]	Brazil	Prospective cohort	*n*: 29 Mean (SD) age: 54.1 (11.5) Males (*n*): 23 Females (*n*): 6	Mean (SD): Time 1: 2.4 (1.2) days Time 2: 8.1 (2.8) days	3-day food diary
Richardson et al., 2001 [3]	UK	Prospective cohort	*n*: 23 Mean (SD) age: 53.9 (1.9) Males (*n*): 10 Females (*n*): 13	3, 6 and 12 months	3-day food diary
Rodrigues et al., 2019 [46]	Brazil	Cross-sectional	*n*: 20 Mean (SD) age: 50 (3) Males (*n*): 14 Females (*n*): 6	Mean (SD): 26 (2) months	3-day food diary

* Studies presented in alphabetical order. CIMT: carotid intima-media thickness; IQR: interquartile range; SD: standard deviation.

**Table 2:** Differences in nutrient intakes between different post-transplant periods: meta-regression analysis of a total of 19 studies.

Nutrient Reference Period *	Comparand *	Coefficient	95% CI		*p*-Values	I^2^
**Energy (kcal)**						
<1 month	1 to <6 months	608.9	292.0	925.8	0.002	63.0
	6 to <12 months	251.3	100.3	402.4	0.003	74.2
	≥12 months	135.1	23.8	246.5	0.021	95.1
1 to <6 months	6 to <12 months	−110.1	−330.8	110.5	0.307	62.1
	≥12 months	−103.7	−224.8	17.4	0.089	93.4
6 to <12 months	≥12 months	−101.5	−311.8	108.9	0.328	92.9
**Protein (% energy)**						
<1 month	1 to <6 months	−1.9	−5.8	1.9	0.283	87.2
	6 to <12 months	−1.1	−2.8	0.5	0.160	86.3
	≥12 months	−0.7	−1.7	0.3	0.163	86.2
1 to <6 months	6 to <12 months	−0.3	−3.1	2.4	0.813	88.9
	≥12 months	−0.1	−1.3	1.2	0.940	88.7
6 to <12 months	≥12 months	0.2	−2.0	2.4	0.843	88.1
**Carbohydrate (% energy)**						
<1 month	1 to <6 months ^†^					
	6 to <12 months	0.7	−4.5	6.0	0.748	77.7
	≥12 months	1.0	−1.2	3.2	0.338	79.9
1 to <6 months	6 to <12 months	1.0	−5.6	6.7	0.659	73.2
	≥12 months	0.4	−1.3	2.0	0.630	76.1
6 to <12 months	≥12 months	1.5	−2.1	5.0	0.399	79.0
**Fat (% energy)**						
<1 month	1 to <6 months	0.1	−1.3	1.6	0.748	79.3
	6 to <12 months ^†^					
	≥12 months	0.2	−4.1	4.6	0.659	75.4
1 to <6 months	6 to <12 months	1.8	−3.0	6.6	0.630	81.9
	≥12 months	0.4	−1.4	2.1	0.399	81.6
6 to <12 months	≥12 months	−1.1	−4.2	2.0	0.748	76.4
**Saturated fat (% energy)**						
<1 month	1 to <6 months †					
	6 to <12 months †					
	≥12 months †					
1 to <6 months	6 to <12 months †					
	≥12 months	1.9	−0.3	4.0	0.087	96.6
6 to <12 months	≥12 months	−0.3	−3.1	2.4	0.781	86.5

* Intakes of each nutrient were compared between two periods: those during the comparand period, compared with those during the reference period. The number of studies varied according to the analysis, as seen in the main figures. † Meta-regression was not performed because of the limited sample size (*n* observations < 10).

**Table 3:** Differences in nutrient intakes by study-level variables: meta-regression analysis.

Study-Level Variable *	Coef	95% CI		*p*-Values	I^2^
**Aetiology—proportion with ARLD (15 studies)**					
Energy (kcal)	−1.0	−7.5	5.4	0.746	89.9
Protein (% energy)	0.0	0.0	0.1	0.184	85.4
Carbohydrate (% energy)	0.0	−0.1	0.1	0.512	67.8
Fat (% energy)	0.0	−0.1	0.1	0.736	77.5
Saturated fat (% energy)					
**Average age (18 studies)**					
Energy (kcal)	49.7	9.0	90.4	0.018	88.1
Protein (% energy)	0.0	−0.4	0.4	0.901	87.5
Carbohydrate (% energy)	−0.5	−1.2	−0.2	0.148	78.7
Fat (% energy)	0.5	−0.1	1.1	0.083	74.2
Saturated fat (% energy)	0.0	−0.4	0.4	0.988	85.0
**Sex—proportion male (18 studies)**					
Energy (kcal)	−0.6	−7.2	6.0	0.848	92.2
Protein (% energy)	0.0	−0.0	0.1	0.237	88.2
Carbohydrate (% energy)	−0.1	−0.1	0.0	0.083	73.0
Fat (% energy)	0.0	−0.1	0.1	0.948	77.2
Saturated fat (% energy)	0.2	0.1	0.3	0.001	86.8
**Year of publication (19 studies)**					
Energy (kcal)	−20.9	−40.2	−1.7	0.034	89.9
Protein (% energy)	0.3	0.1	0.4	<0.001	87.4
Carbohydrate (% energy)	0.0	−0.3	0.3	0.923	79.1
Fat (% energy)	−0.3	−0.5	−0.1	0.014	75.0
Saturated fat (% energy)	−0.1	−0.9	0.7	0.798	98.8
**Continent—Europe vs. other (19 studies)**					
Energy (kcal)	200.6	7.6	393.6	0.042	88.7
Protein (% energy)	−2.5	−4.1	−0.9	0.004	88.2
Carbohydrate (% energy)	2.3	−0.6	5.2	0.110	73.9
Fat (% energy)	3.7	1.2	6.2	0.005	73.3
Saturated fat (% energy)	1.1	−2.6	4.7	0.528	98.9
**Dietary assessment method—food diary vs. other (19 studies)**					
Energy (kcal)	−41.4	−144.0	61.3	0.418	92.4
Protein (% energy)	−0.4	−1.3	0.5	0.395	88.5
Carbohydrate (% energy)	−0.3	−1.8	1.3	0.746	79.0
Fat (% energy)	0.5	−1.0	1.9	0.520	78.8
Saturated fat (% energy)	2.3	−0.2	4.7	0.065	97.6

* Each study-level variable was used as an independent variable in a meta-regression, in which each nutrient intake type was modelled as a dependent variable. The units or scales were 0 to 1 for proportions, years for age and publications, and 0 or 1 for continent and dietary assessment method. CI: Confidence interval; Coef: coefficient; ARLD: alcohol-related liver disease.

## Data Availability

The data that support the findings of this study are available from the corresponding author upon reasonable request.

## Supplementary Materials

The following supporting information can be downloaded at: https://www.mdpi.com/article/10.3390/nu15112487/s1, Figure S1: Mean daily protein intake (percent energy) with 95% confidence intervals from the included studies, categorised by time post-transplant; Figure S2: Mean daily carbohydrate intake (percentage of energy) with 95% confidence intervals from the included studies, categorised by time post-transplant; Figure S3: Mean daily fibre intake (grams) with 95% confidence intervals from the included studies, categorised by time post-transplant; Figure S4: Mean daily total fat intake (percentage of energy) with 95% confidence intervals from the included studies, categorised by time post-transplant; Figure S5: Mean daily saturated fat intake (percentage of energy) with 95% confidence intervals from the included studies, categorised by time post-transplant; Figure S6: Mean daily energy intake (kcal/day) with 95% confidence intervals from the included studies with a low risk of bias, categorised by time post-transplant; Figure S7: Mean daily protein intake (percentage of energy) with 95% confidence intervals from the included studies with a low risk of bias, categorised by time post-transplant; Figure S8: Mean daily carbohydrate intake (percentage of energy) with 95% confidence intervals from the included studies with a low risk of bias, categorised by time post-transplant; Figure S9: Mean daily fibre intake (grams) with 95% confidence intervals from the included studies with a low risk of bias, categorised by time post-transplant; Figure S10: Mean daily fat intake (percentage of energy) with 95% confidence intervals from the included studies with a low risk of bias, categorised by time post-transplant; Figure S11: Mean daily saturated fat intake (percentage of energy) with 95% confidence intervals from the included studies with a low risk of bias, categorised by time post-transplant; Table S1: Search terms and example of a search using Embase; Table S2: Findings from four studies reporting food groups; Table S3: Findings from nine studies comparing the nutritional intake at two or more time points after liver transplant, with different anti-rejection drugs and between sexes; Table S4: Meta-regression sensitivity analysis including studies with a low risk of bias to investigate the differences in nutrient intake for the study-level variables; Table S5: Meta-regression sensitivity analysis comparing converted data with non-converted data.
