# Supplementary material for: Nutritional Intake after Liver Transplant: Systematic Review and Meta-Analysis
Source: Nutrients. 2023 May 26;15(11):2487. doi: 10.3390/nu15112487 (PMC10255417; doi:10.3390/nu15112487)
Supplement: Supplementary file 1 [file nutrients-15-02487-s001.zip › nutrients-2418022-supplementary.pdf]

Supplementary Figures

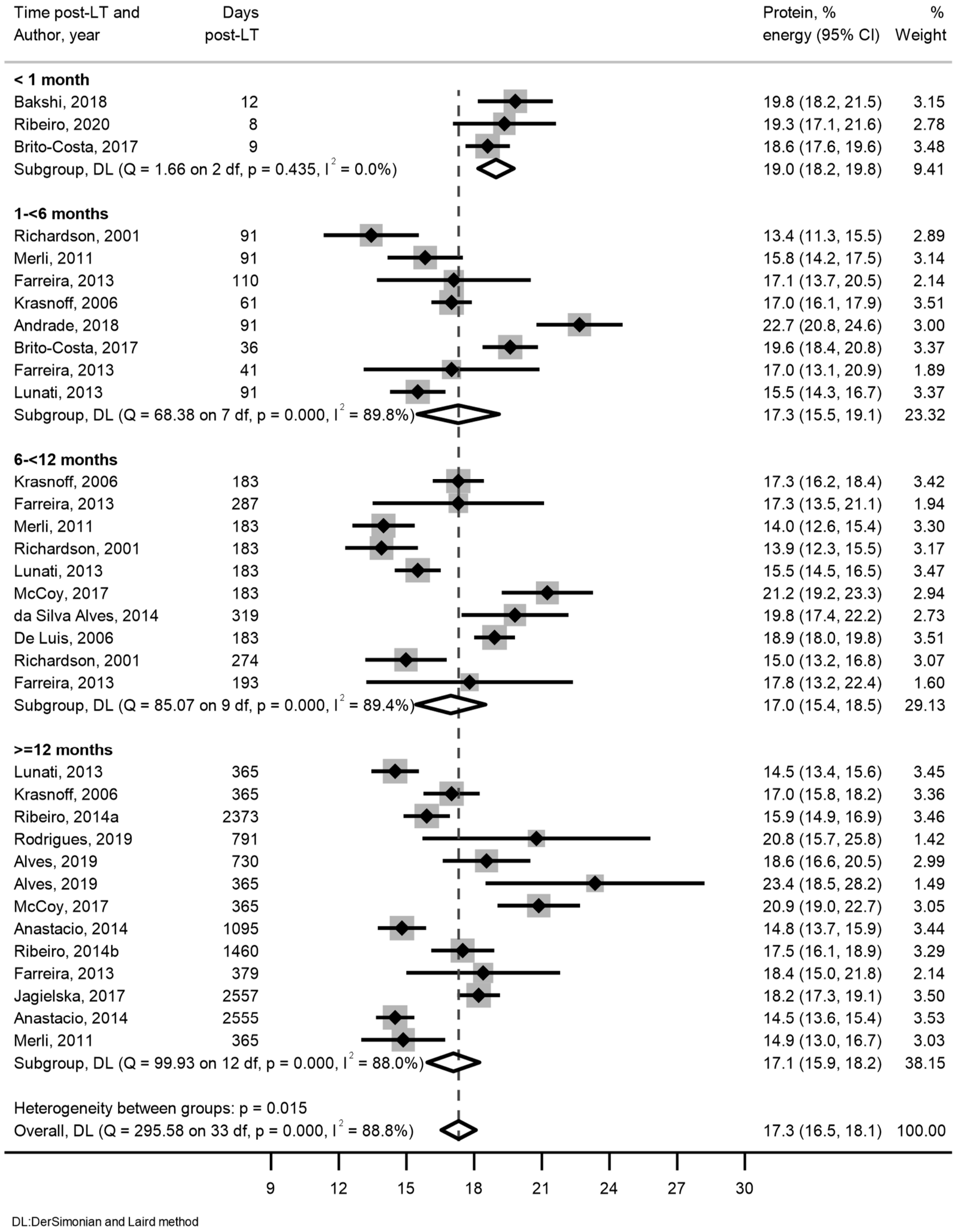

Figure S1: Mean daily protein intake (percent energy) with 95% confidence intervals from included studies categorised by time post-transplant.

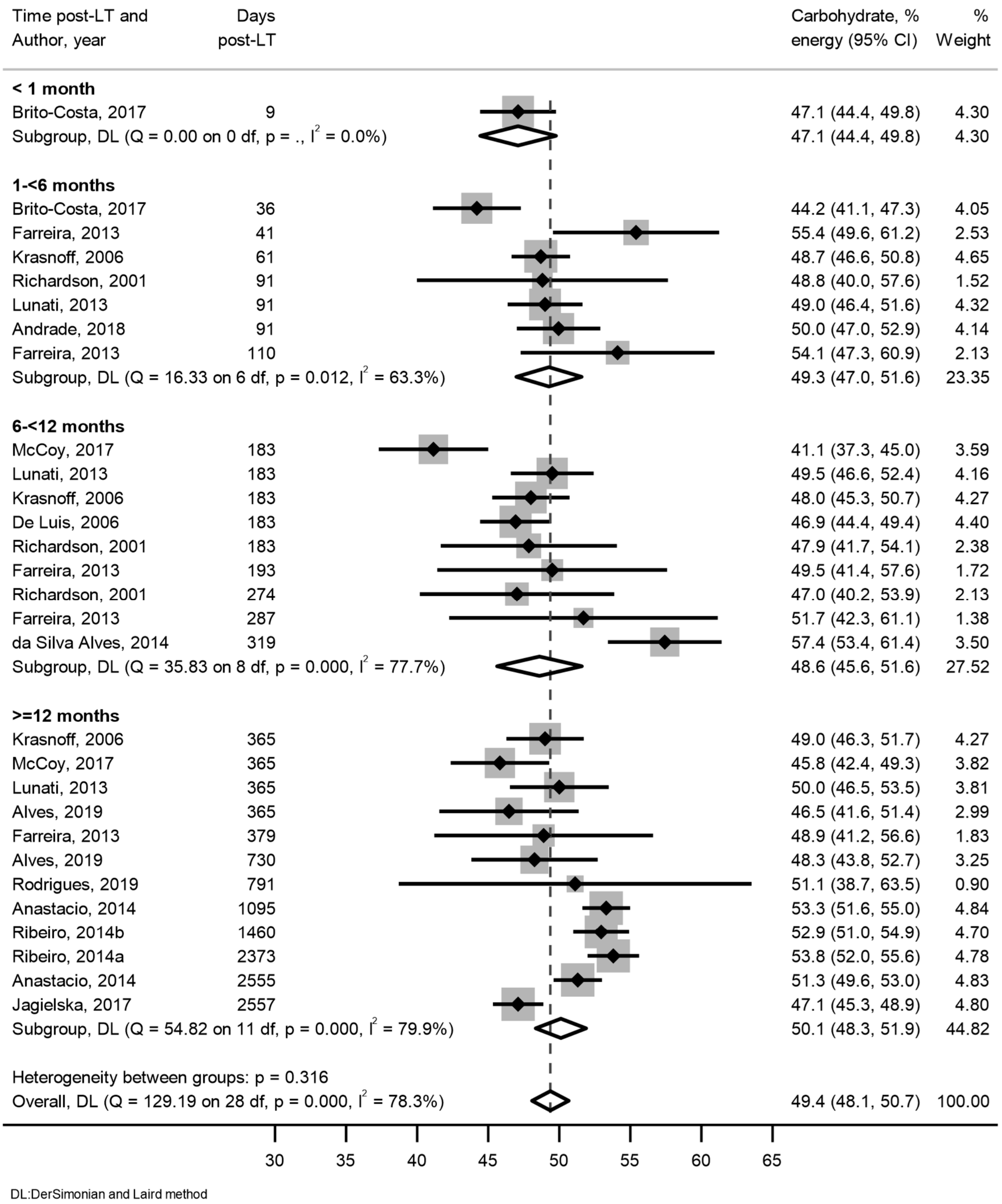

Figure S2. Mean daily carbohydrate intake (percent energy) with 95% confidence intervals from included studies categorised by time post-transplant.

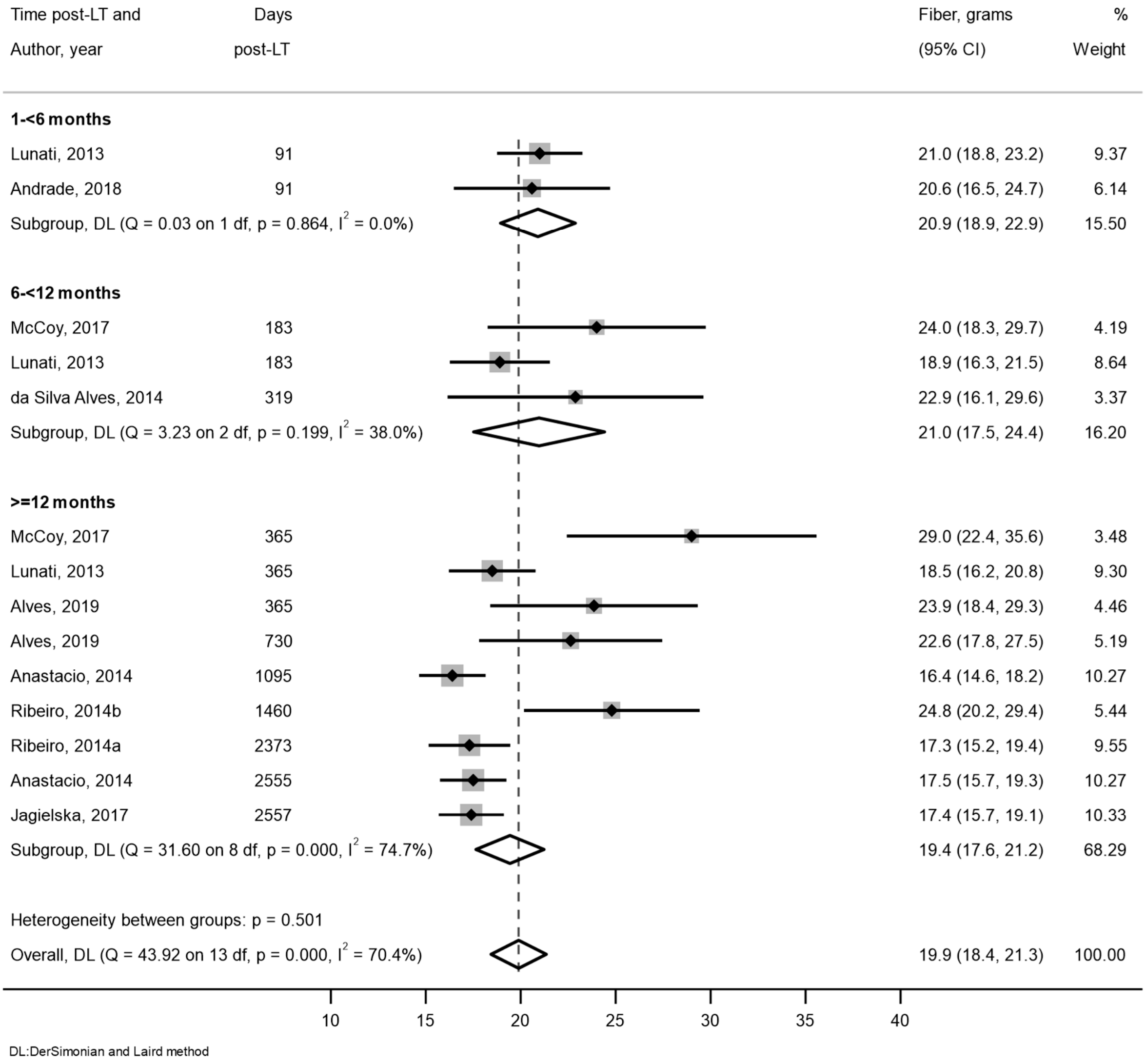

Figure S3. Mean daily fiber intake (grams) with 95% confidence intervals from included studies categorised by time post-transplant.

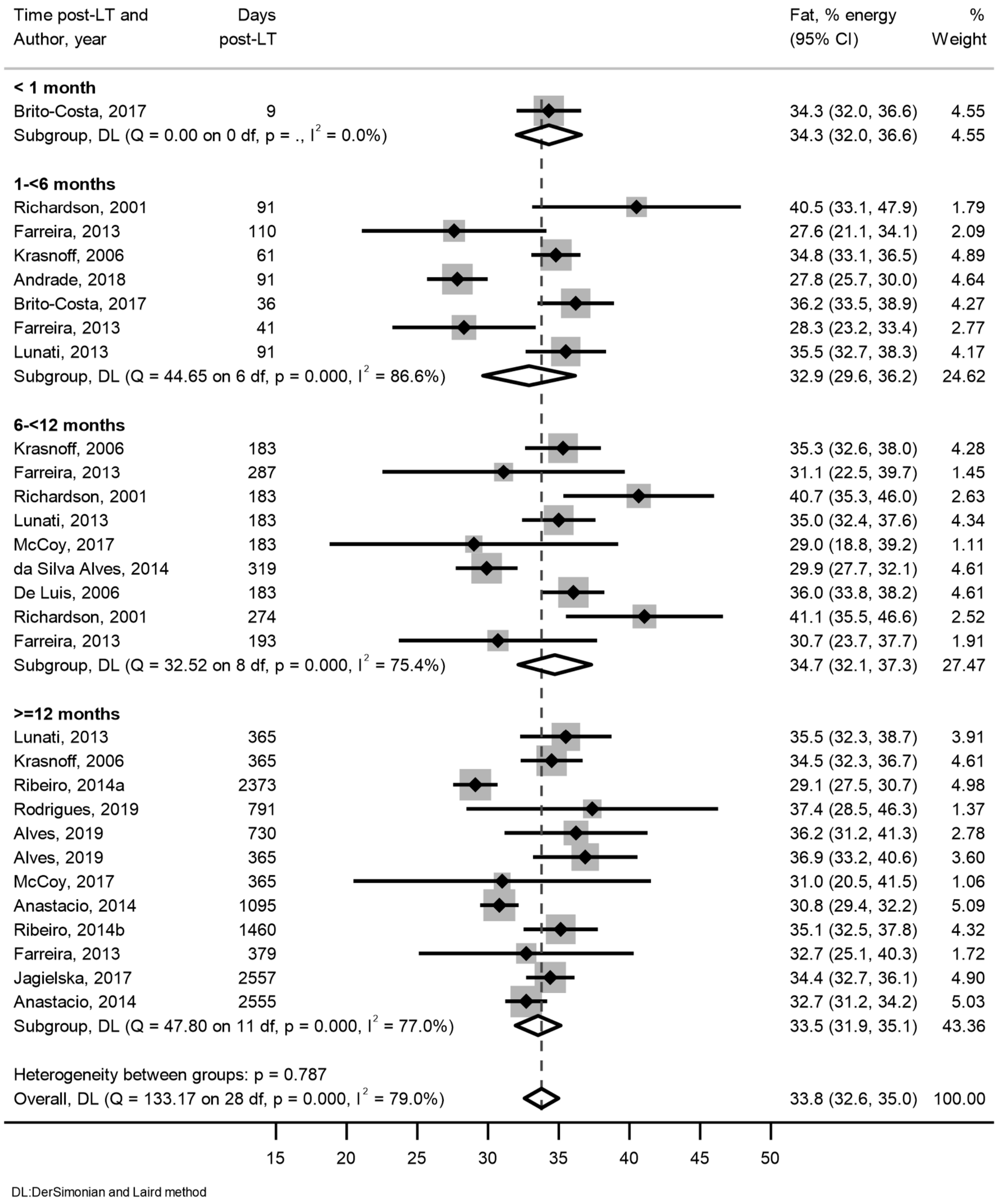

Figure S4. Mean daily total fat intake (percent energy) with 95% confidence intervals from included studies categorised by time post-transplant.

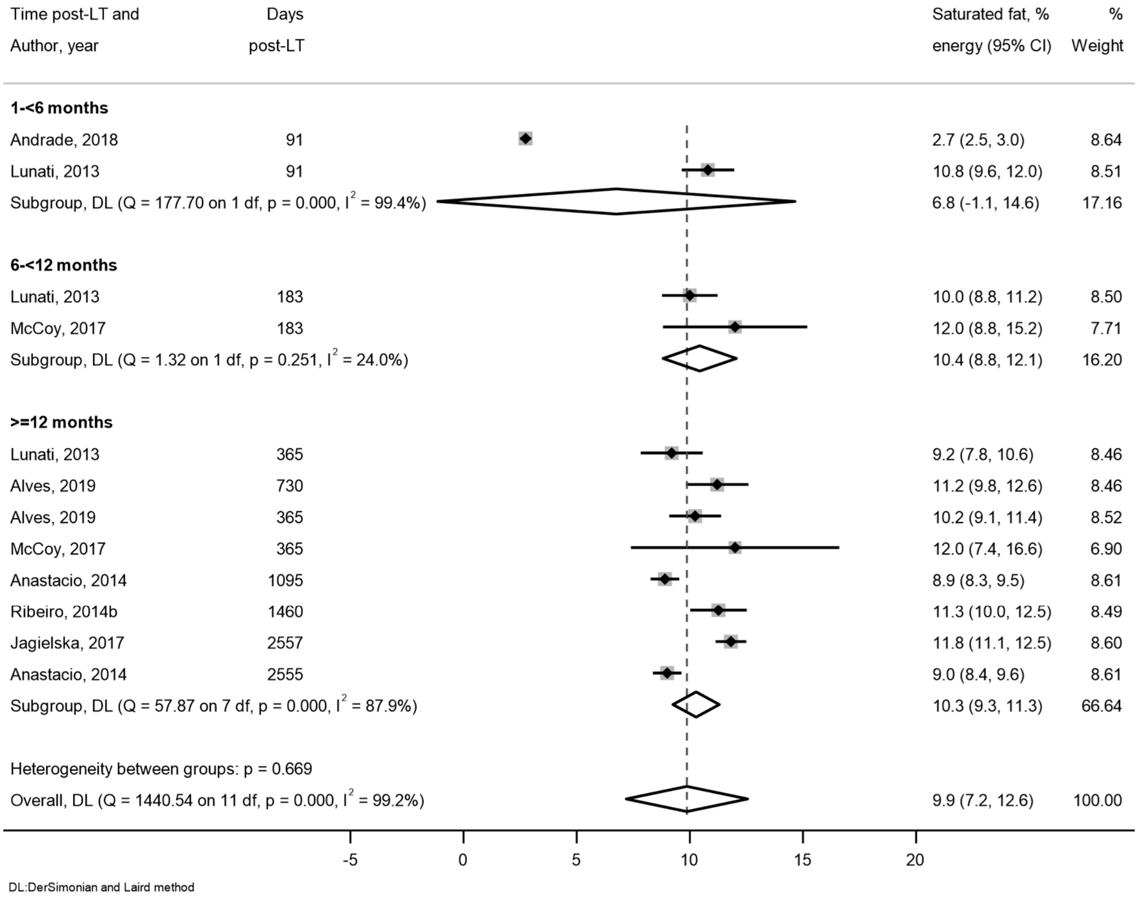

Figure S5. Mean daily saturated fat intake (percent energy) with 95% confidence intervals from included studies categorised by time post-transplant.

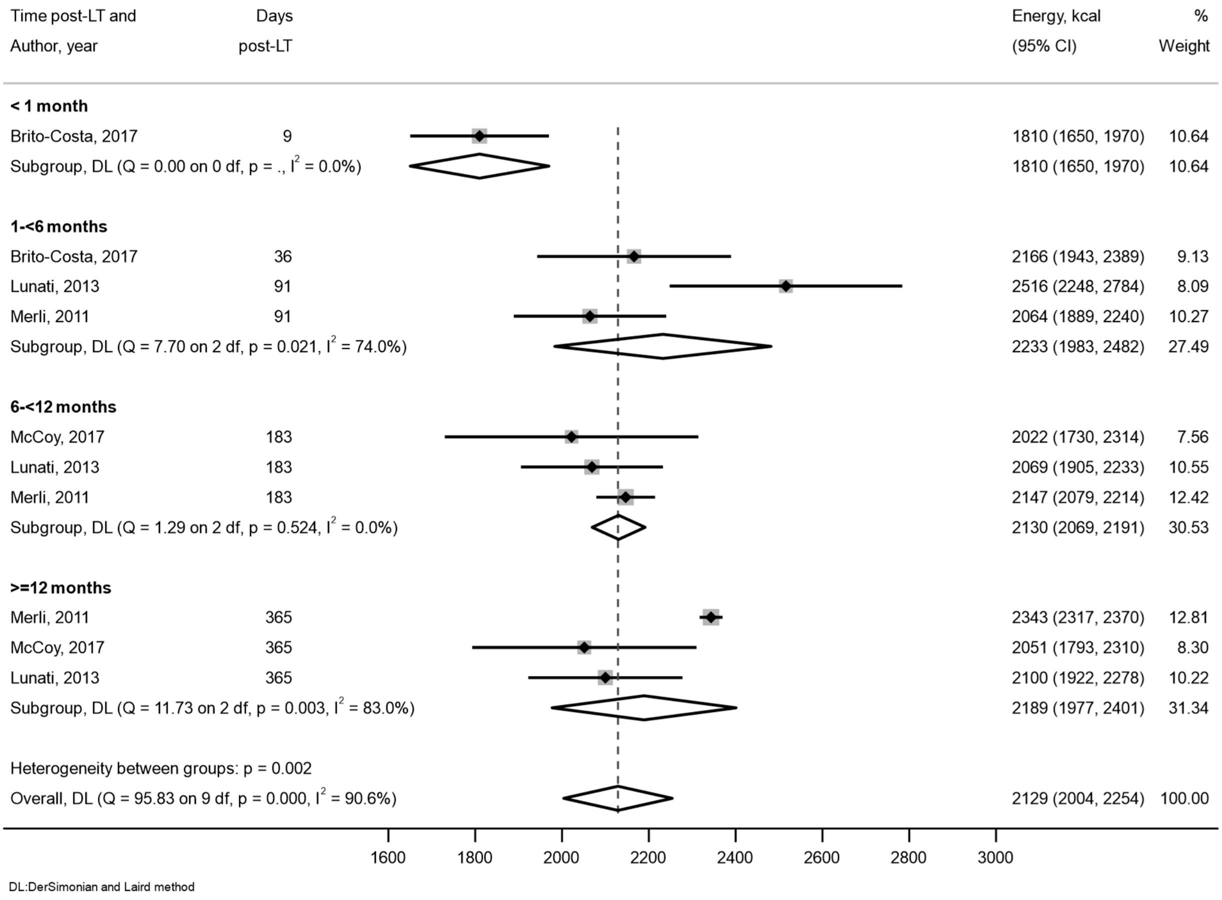

Figure S6. Mean daily energy intake (kcal/day) with 95% confidence intervals from included studies with low risk of bias categorised by time post-transplant.

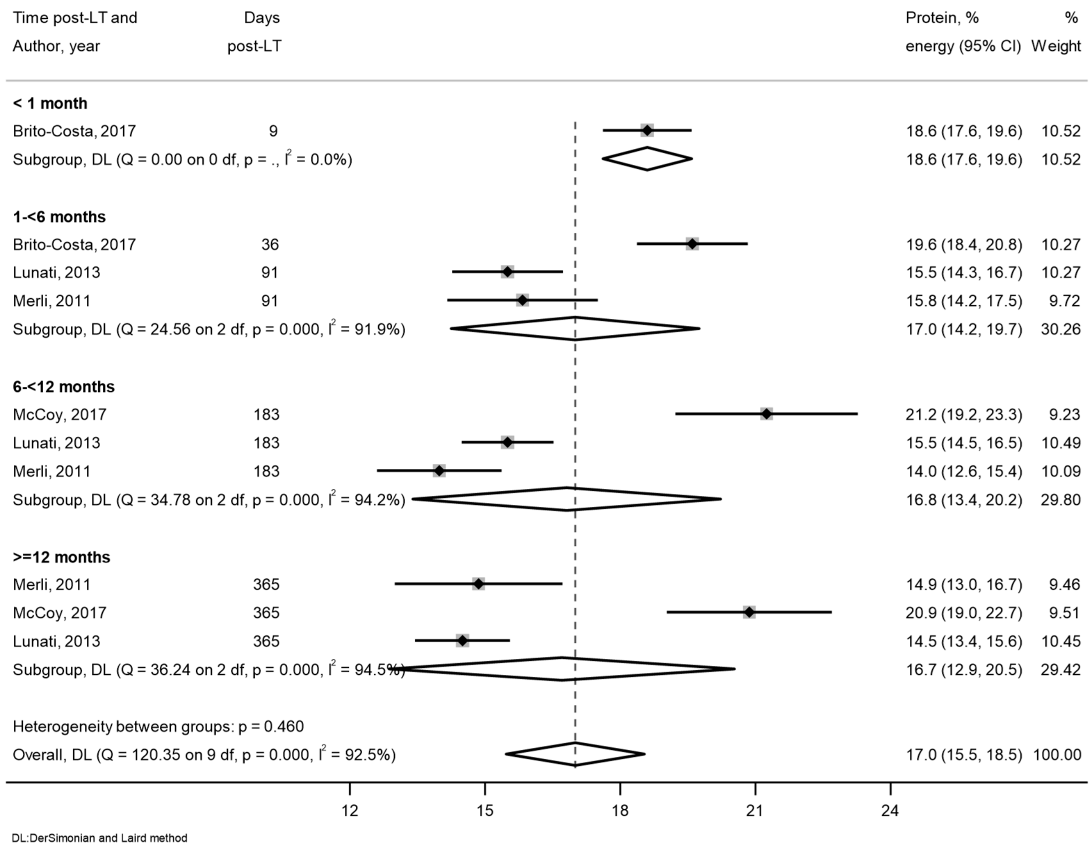

Figure S7. Mean daily protein intake (percent energy) with 95% confidence intervals from with low risk of bias categorised by time post-transplant.

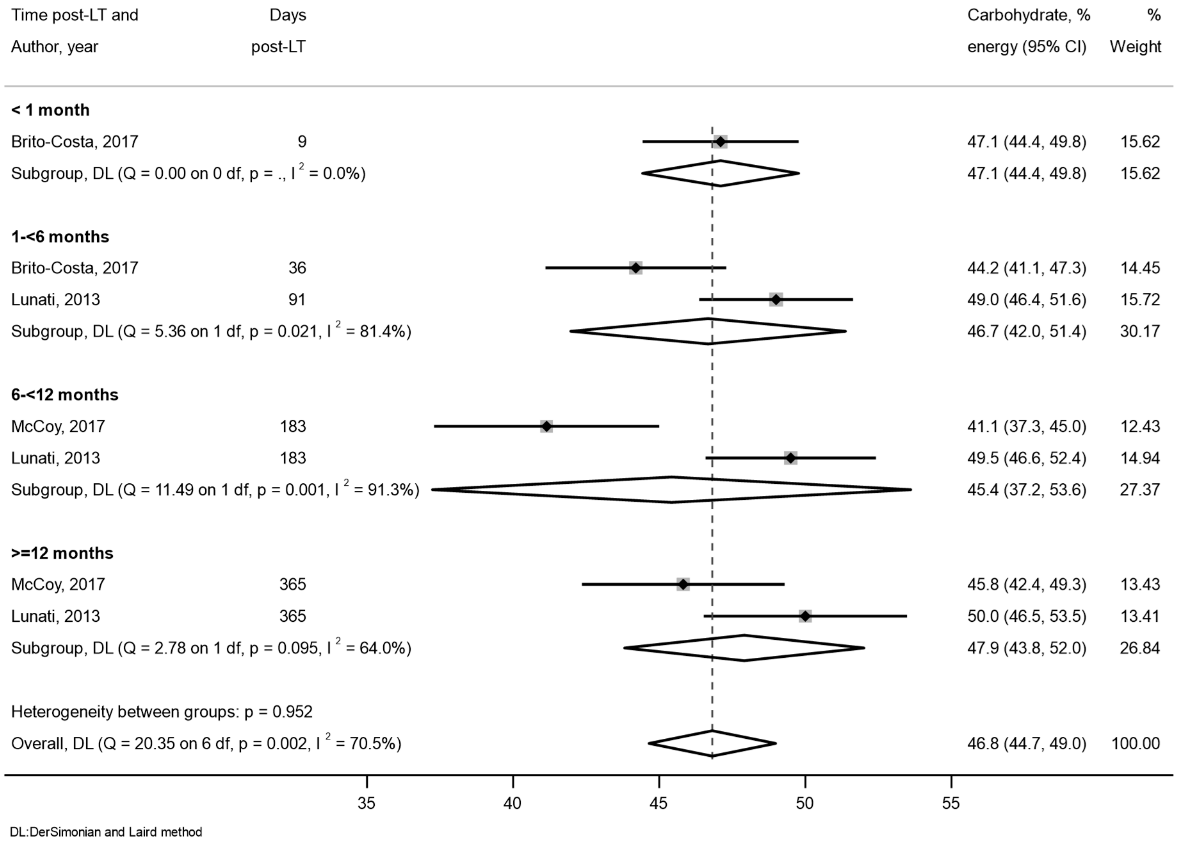

Figure S8. Mean daily carbohydrate intake (percent energy) with 95% confidence intervals from with low risk of bias categorised by time post-transplant.

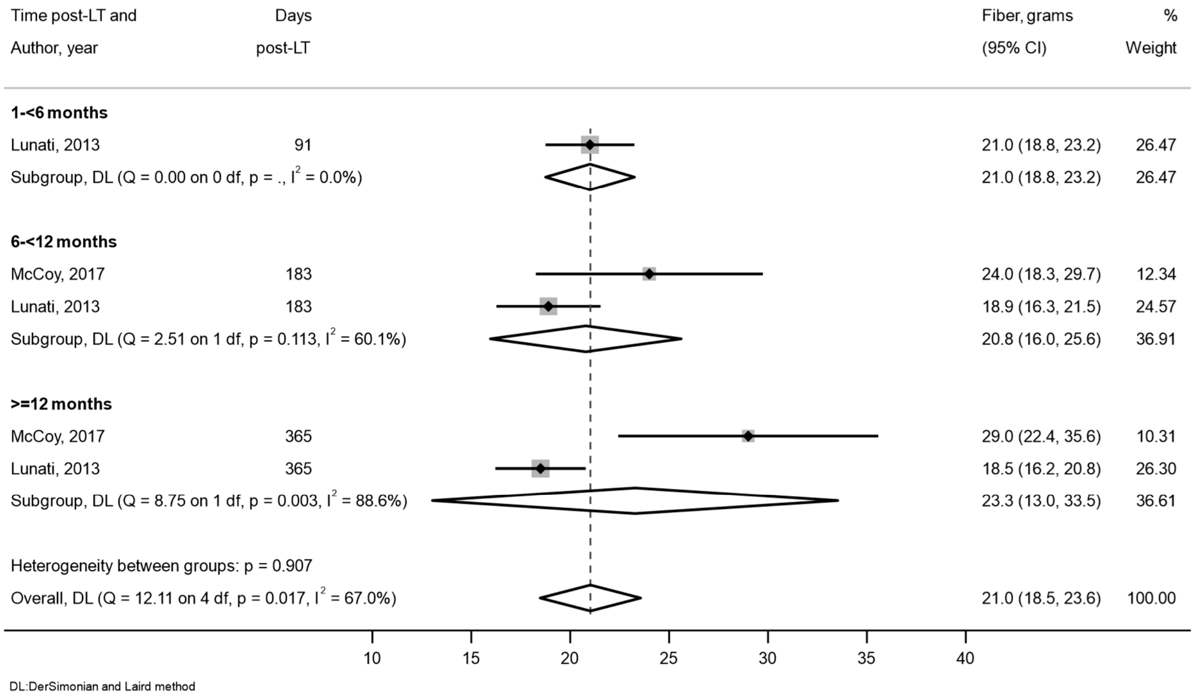

Figure S9. Mean daily fiber intake (grams) with 95% confidence intervals from with low risk of bias categorised by time post-transplant.

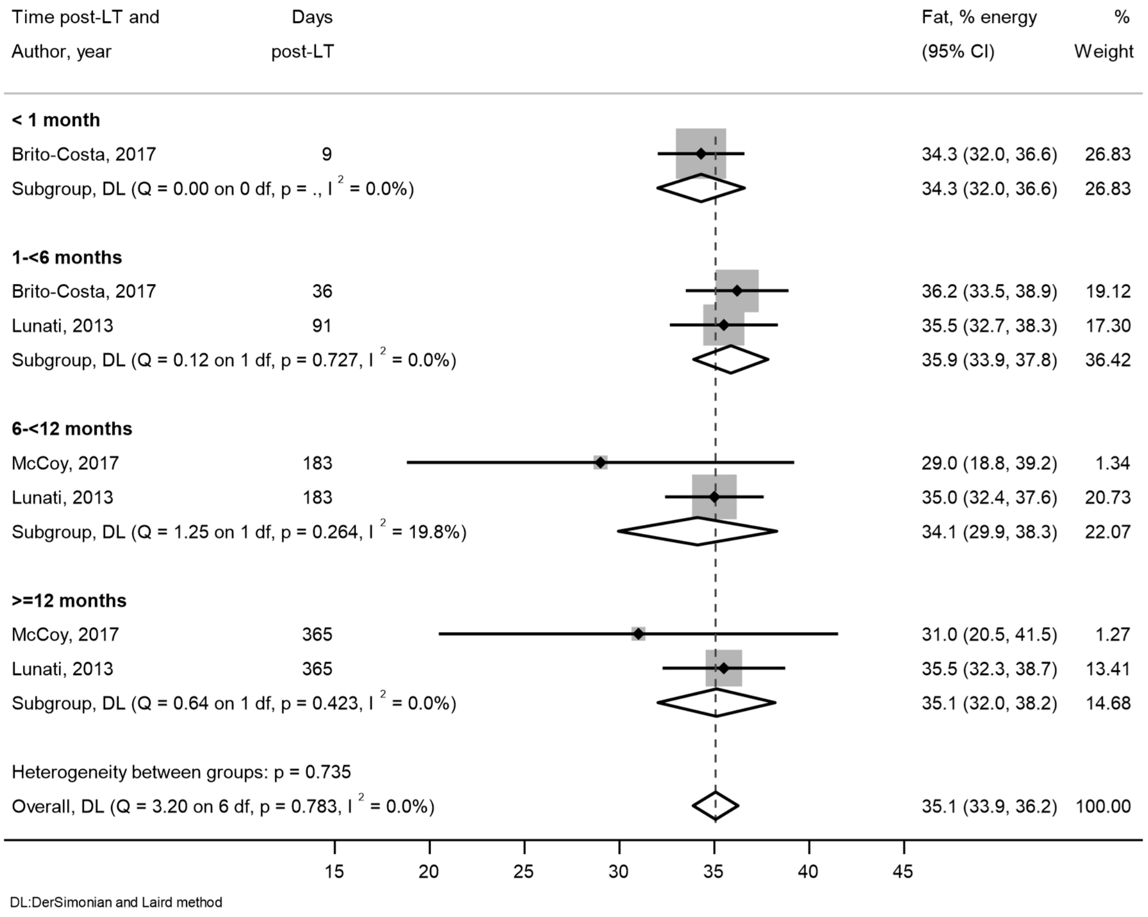

Figure S10. Mean daily fat intake (percent energy) with 95% confidence intervals from with low risk of bias categorised by time post-transplant.

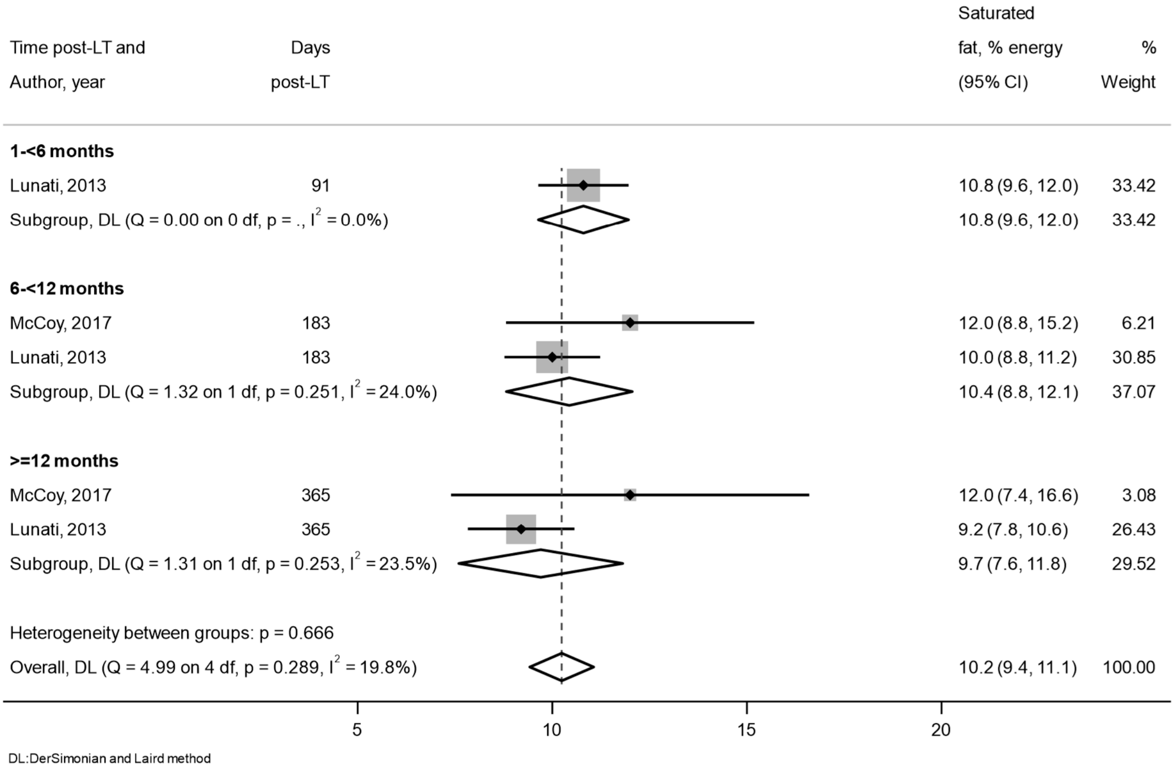

Figure S11. Mean daily saturated fat intake (percent energy) with 95% confidence intervals from with low risk of bias categorised by time post-transplant.

Supplementary Tables

Table S1. Search terms and example of search using Embase

**Population**		**Outcome**	
**Text words**	**MESH terms**	**Text words**	**MESH terms**
"Liver transplant*"	Liver graft/	Diet*	diet/
"Hepatic transplant*"	Liver transplantation/	nutrition*	nutrition/
"Liver graft*"		food	physical activity/
		beverage*	exercise/
		drink*	lifestyle/
		intake	qualitative research/
		feeding	interview/
		eat*	
		meal*	
		lifestyle*	
		exercis*	
		"physical activit*"	
		mobil*	
		ambulat*	
		"physical exertion"	
		sport*	
		"bodily movement"	
		"motor activit*"	
		"physical effort"	
		"Lived experience*"	
		Qualitative	

Example of search using Embase

1. "liver transplant*".ti,ab.
2. "hepatic transplant*".ti,ab.
3. "liver graft*".ti,ab.
4. exp liver graft/ (Subject heading term)
5. 1 or 2 or 3 or 4
6. Diet*.ti,ab.
7. nutrition*.ti,ab.
8. food.ti,ab.
9. beverage*.ti,ab.
10. drink*.ti,ab.
11. intake.ti,ab.
12. feed*.ti,ab.
13. eat*.ti,ab.
14. meal*.ti,ab.
15. lifestyle*.ti,ab.
16. exercis*.ti,ab.
17. "physical activit*".ti,ab.
18. mobil*.ti,ab.
19. ambulat*.ti,ab.
20. "physical exertion".ti,ab.
21. "bodily movement".ti,ab.
22. "motor activit*".ti,ab.
23. "physical effort".ti,ab.
24. "lived experience".ti,ab.
25. qualitative.ti,ab.
26. exp diet/
27. exp nutrition/
28. exp physical activity/

29. exp exercise/
30. exp lifestyle/
31. exp qualitative research/
32. exp interview/
33. sport*.ti,ab.
34. 6 or 7 or 8 or 9 or 10 or 11 or 12 or 13 or 14 or 15 or 16 or 17 or 18 or 19 or 20 or 21 or 22 or 23 or 24 or 25 or 26 or 27 or 28 or 29 or 30 or 31 or 32 or 33
35. 5 and 34

Table S2. Findings from four studies reporting food groups*

Author, year	**Bakshi and Singh., 2018**<br><br>**Mean (SD)**	**McCoy et al., 2017**<br><br>**Median (IQR)**		**Ribeiro et al., 2014**<br><br>**Median (range)**	**Anastacio et al., 2013**<br><br>**Median (IQR)**
Time post-transplant	12 days	6 months	12 months	Mean (SD): 4 (3) years	Median (range); 4 years (13 months – 14 years)
Cereals, bread, etc. (g)/ Cereal grains and products (g)/ Cereals and cereal products (g)/ Breads, cereals, tubers (g)	182.2 (80.9)	154 (84-200)	200 (150-285)	390 (89-1680)	379 (255-550)
Cereal-based products and dishes (g)		68 (42-136)	122 (55-195)		
Wholegrain cereals (g)				0 (0-190)	
Vegetables (g)/ Other vegetables (g)	88.4 (101.6)	199 (89-319)	234 (134-344)	140 (0-600)	104 (65-200)
Leafy vegetables (g)/ Greenery (g)	1.1 (8.2)			110 (0-521)	
Roots and tubers (g)	4.8 (15.8)				
Fruit (g)	10.8 (42.5)	185 (58-303)	184 (49-333)	123 (0-717)	123 (45-248)
Milk (mL)				150 (0-720)	125 (0-200)
Cheese (g)				0 (0-15)	10 (0-15)
Yogurt (g)/ (ml)				0 (0-150)	0 (0-0)
Milk and milk products (g/ml)/ Dairy products and dishes (g)	605.4 (224.4)	264 (153-642)	418 (213-721)	150 (0-720)	
Egg products and dishes (g)		0 (0-31)	0 (0-11)		
Beans (g)/ Pulses and legumes (g)	48.3 (63.4)				120 (60-200)
Meat, poultry, etc. (g)	168.3 (177.6)	154 (96-197)	128 (107-196)	127 (0-500)	120 (91-185)
Fish and seafood products and dishes (g)		21 (0-29)	21 (2-48)		
Sweet beverages (mL)[28]/ Sugar sweetened beverages (g)				200 (0-2000)	154 (0-385)
Sugar and sweets (g)	6 (4.6)			0 (0-500)	20 (0-30)
Fat and oil (g)	9.3 (10.9)	10 (1-17)	10 (5-19)		19 (8-28)

*Studies reported in order of time post-transplant. IQR: interquartile range; SD: standard deviation.

Table S3: Findings from nine studies comparing nutritional intake at two or more time points after liver transplant, with different anti-rejection drugs and between sexes.

Correlate	Author	Measure	Statistical analysis method reported	Energy	Protein	Carbohydrate	Fiber	Fat	Sat fat
Time: 2.4 days vs. 8.1 days	Ribeiro et al., 2020	Median [IQR]	Kruskal-Wallis test and Dunn post test*	↑594.9 [398.9-989.7] vs 1251.8 [1029.2-1779.1] kcal, p<0.05	↑27.1 [13.3-47.7] vs. 63.4 [46.3-93.8] grams, p<0.05<br>18.6 [14.3–19.9] vs. 18.4 (15.6–23.9) percent energy, p>0.05				
Time: Day 9 vs. Day 12	Bakshi et al., 2018	Mean±SD	ANOVA followed by post hoc test	↑Post-hoc: 1285 ± 594, vs. 1485 ± 732 kcal, p=0.019	↑Post-hoc: 63.5 ± 31.7, vs. 73.6 ± 36.1 grams, p=0.014				
Time: Day 9 vs. Day 35	Brito costa et al., 2016 and Brito costa et al., 2017	Mean±SD	Friedman test, multiple comparisons	↑1810.0±455.1 vs. 2166±635.4 kcal, p<0.05	18.6±2.8 vs. 19.6±3.5 percent energy, p>0.05.	↓47.1±7.6 vs. 44.2±8.8 percent energy, p<0.05		34.3±6.5 vs. 36.2±7.7 percent energy, p>0.05	
Time:<br>41 days<br>110 days<br>182 days<br>287 days<br>379 days	Farreira et al., 2013	Mean±SD	Repeated-measures analyses of variance asphericity correction, followed by paired t tests with Bonferroni correction*	No statistically significant differences reported**	No statistically significant differences reported**	Post-hoc:<br>↓55.4 ± 6.0 percent energy at 110 days vs. 51.7 ± 9.7 percent energy at 287 days, p<0.01<br>↓55.4 ± 6.0 percent energy at 110 days vs. 48.9 ± 7.9 percent energy at 379 days, p<0.01		No statistically significant differences reported**	
Time: 6 and 12 months	McCoy et al., 2017	Mean±SD	ANOVA followed by post-hoc Tukey's test*	9.444±2.535 at pre-transplant, 8.460±1.917 at 6 months post-transplant, and 8.583±1.645 kilojoules at 12 months, p=0.415.	1.7±0.5 at pre-transplant, 1.6±0.4 at 6 months post-transplant, and 1.6±0.5 grams/kilogram at 12 months post-transplant, p=0.590.	Post-hoc:<br>↑208±49 grams at 6 months post-transplant vs.235±36 at 12 months post-transplant, p<0.05	26±11 at pre-transplant, 24±9 at 6 months post-transplant, and 29±10 grams at 12 months post-transplant, p=0.745.	31±9 at pre-transplant, 29±16 at 6 months post-transplant, 31±16 percent energy at 12 months post-transplant, p=0.511.	32±15 at pre-transplant, 23±23 at 6 months post-transplant, 27±28 grams at 12 months post-transplant, p=0.558.
Time: 3, 6 and 12 months	Merli et al., 2011	Median (range)	ANOVA followed by	29 (23-41) at pre-transplant, 31 (24-41) at 3 months post-	0.9 (0.6-4.4) at-pre-transplant, 1.2 (0.7-1.9) at 3				

			Bonferroni test*	transplant, 32 (29-37) at 6 months post-transplant, and 33 (27-36) kcal/kilogram at 12 months post-transplant, p=0.0006.<br><br>No statistically significant differences reported between post-transplant time points**	months post-transplant, 1.1 (0.7-1.5) at 6 months post-transplant, and 1.2 (0.7-1.6) g/Kg at 12 months post-transplant, p=0.01.<br><br>No statistically significant differences reported between post-transplant time points**				
Time: 3, 6 and 9 months	Richardson et al., 2001	Mean±SEM	Repeated-measures analysis of variance and for pairwise comparisons paired t tests*	No statistically significant differences between post-transplant time-points reported**	No statistically significant differences between post-transplant time-points reported**	No statistically significant differences between post-transplant time-points reported**		No statistically significant differences between post-transplant time-points reported**	
Time: 3 years vs. 7 years	Anastacio et al., 2014	Mean±SD	Paired t-test	↑1920.9±633.1 vs. 2016.7±666.1 kcal, p<0.01.	↓14.8±4.4 vs. 14.5±3.5 percent energy, p<0.01.	53.3±6.9 vs. 51.3±7.0 percent energy, p=0.46.	↑16.4±7.2 vs. 17.5±7.2 grams, p<0.01.	30.8±5.6 vs. 32.7±6.1 percent energy, p=0.9.	8.9 ± 2.6 vs. 9.0 ± 2.6 percent energy, p=0.47.
Immunosuppression: Cyclosporine vs. Tacrolimus	Brito costa et al., 2016	Mean±SD	Not reported	2083.8±637.9 vs. 2049.3±895.1kcal, p=0.756	17.8±5.1 vs. 18.8±5.6 percent energy, p=0.465	50.4±9.5 vs. 50.3±10 percent energy, p=0.808		31.8±9.2 vs.30.9±7.2 percent energy, p=0.834	
Sex: Female vs. Male	Jagielska et al., 2017	Proportion	Not reported	100.0% vs. 93.6% consuming <estimated requirements, p=0.3675	25% vs. 6.5% consuming <15% energy from protein, p=0.0887	25% vs. 74.2% consuming <50% energy from carbohydrate, p=0.0031	8.3% vs. 9.7% consuming >25g fiber, p=0.8917<br><br>0% vs. 6.5% consuming >30g fiber, p=0.3675	41.7% vs. 9.7% consuming <30% energy from fat, p=0.0156	41.7% vs. 16.1% consuming <10% energy from saturated fat, p=0.0002

Significant findings in gray; * including pre- and post-transplant measures; ** no p values or confidence intervals reported; ↑ Intake significantly increased as time progressed post-transplant; ↓ Intake significantly decreased as time progressed post-transplant; blank cells show variable not investigated. IQR: interquartile range; SD: standard deviation.

Table S4. Meta-regression sensitivity analysis including studies with low risk of bias to investigate differences in nutrient intake for study level variables* (n=10).

	Coefficient	95% CI		p	I^2
**Average age**					
Energy (kcal)	102.5	-141.6	346.6	0.361	85.63
Protein (% energy)	-2.5	-6.0	0.9	0.129	91.44
**Sex**					
Energy (kcal)	-19.7	-44.4	5.0	0.102	87.13
Protein (% energy)	0.4	0.2	0.7	0.004	76.17
**Year of publication**					
Energy (kcal)	-35.1	-83.0	12.7	0.129	84.69
Protein (% energy)	0.9	0.6	1.2	<0.001	59.83
**Continent – Europe vs. other**					
Energy (kcal)	108.6	-279.3	496.4	0.537	91.04
Protein (% energy)	-5.0	-8.7	-1.3	0.014	89.65
**Dietary assessment method – food diary vs. other**					
Energy (kcal)	68.2	-106.9	243.4	0.395	86.41
Protein (% energy)	-0.5	-3.1	2.2	0.696	92.32

* Meta-regression was not performed for different time periods post-transplant or etiology of liver disease due to the limited number of observations for categories (n studies in total <10).

Table S5. Meta-regression sensitivity analysis comparing converted with non-converted data*

Variable	No. observations	Coefficient	95% CI		p	I^2
Energy (kcal)	35	-18.8	-241.1	203.5	0.864	88.95
Protein (% energy)	33	0.3	-1.4	2.1	0.692	88.60
Carbohydrate (% energy)	29	-1.3	-4.3	1.6	0.365	78.87
Fat (% energy)	29	3.4	0.6	6.2	0.018	77.76
Saturated fat (% energy)	12	-0.7	-4.3	2.9	0.679	98.96
Fiber (grams)	14	3.9	-0.2	8.1	0.058	95.74

* Conversion means, for example, a statistical conversion from medians + interquartile range to means + standard deviation.
